# Prevention of umbilical outpouchings and mortality in pigs: Meloxicam, tying, cutting, and chlorhexidine versus amoxicillin or no treatment? A clinical field trial

**DOI:** 10.1186/s40813-024-00358-w

**Published:** 2024-02-16

**Authors:** Marie-Louise Hansen, Inge Larsen, Tina Birk Jensen, Charlotte Sonne Kristensen, Ken Steen Pedersen

**Affiliations:** 1 Department of Veterinary and Animal Sciences, Faculty of Health and Medical Sciences, University of Copenhagen, Grønnegårdsvej 2, 1870 Frederiksberg C, Denmark; 2 SEGES Innovation, Agro Food Park 15, 8200 Aarhus, Denmark; 3 Ø-Vet A/S, Køberupvej 33, 4700 Næstved, Denmark

**Keywords:** Umbilical outpouchings, Pigs, Prevention, NSAID, Antibiotics, Disinfection, Amoxicillin, Meloxicam, Chlorhexidine

## Abstract

**Background:**

Umbilical outpouchings (UOs) are common in Danish pigs. Neonatal antibiotics are therefore used with the hope of reducing umbilical infections and subsequently UOs. However, the effect of neonatal antibiotics on preventing UO has been the subject of mixed conclusions, and secondly, treating all animals with antibiotics might exacerbate the development of antimicrobial resistance.

This study analysed the effects of different treatments on the prevalence of umbilical outpouchings and mortality from birth to nursery unit. All treatment was on the day of birth. The groups were: a negative control group, an antibiotic group receiving amoxicillin, and an experimental group where the piglets had their umbilical cord disinfected with chlorhexidine, followed by tying and clipping, and lastly, injection with meloxicam. The pigs were examined six weeks after weaning, and all pigs that died during the study were autopsied.

**Results:**

There were 5494 pigs divided across the three groups. There were no statistically significant differences in UO prevalence between the groups: control 3.9%, antibiotic 4.2%, and experimental 4.0% (*p* = 0.87). The only variable affecting the prevalence of UOs in this study was sex with females being at higher risk. There were no statistically significant differences in mortality between the groups from birth until departure from the nursery unit: control 22.9%, antibiotic 21%, and experimental 21.4% (*p* = 0.33). The variables affecting mortality were sex, intrauterine growth restriction (IUGR), birth weight, and cross fostering. Males had higher odds of dying, as had piglets recorded with some degree of IUGR. Also, low birth weight increased the odds of dying for all weight quartiles compared to the fourth (the heaviest piglets > 1.6 kg), as well as cross fostering increased the odds ratio of dying.

**Conclusions:**

This study found no significant differences in the prevalence of UOs and mortality following different treatments at birth. The study showed that the prevalence of UO and mortality was not reduced following the administration of amoxicillin or meloxicam in combination with disinfection and tying of the umbilical cord.

**Supplementary Information:**

The online version contains supplementary material available at 10.1186/s40813-024-00358-w.

## Background

Umbilical outpouchings (UOs) are very common in Danish pigs. Recent research shows prevalences of at least 2.9% in weaners [1] and with a production of 31.8 million pigs [2] close to a million Danish pigs are affected annually. Most pigs born in Denmark are treated systematically with metaphylactic antibiotics at birth [1]. This procedure is under scrutiny because of the growing concerns about antibiotic consumption and a focus on more sustainable pig production. One of the main purposes of neonatal antibiotics is the prevention of umbilical infections hypothesized to contribute to the development of UOs. However, the effect of neonatal antibiotics on preventing UO has been the subject of mixed conclusions in earlier research [3–5], and secondly, treating all animals with antibiotics might exacerbate the development of antimicrobial resistance.

A Californian study from the eighties [3] was unable to show significant differences in the incidence of umbilical hernia between three different groups: One receiving oxytetracycline once at birth, one receiving oxytetracycline at birth, day 5 and 7, and a negative control. The treated groups did have a lower, but insignificant, incidence of umbilical hernia. Despite weekly examinations focusing on hernia development and omphalitis, omphalitis was only found in 0.3% (9/2958) of the piglets.

In a large genetic study (8276 litters) no effect of clipping dried navel cords was demonstrated, nor did administration of long acting ceftiofur at birth reduce hernia prevalence significantly [4]. In contrast to this, a Finnish study from 2017 demonstrated a significant reduction in mortality and hernia prevalence, in pigs that received amoxicillin at birth [5].

Only a few studies have investigated the effect of using antiseptics on the umbilical cord and subsequent outcomes. One American study investigated the effect of three different navel dips and one negative control, on umbilical healing rate and omphalitis within 48 post-partum. The study showed no effect on healing and none of the 470 piglets developed omphalitis [6]. A Danish study examining the effect of autogenous vaccinations and iodine treatment on the risk of developing UOs in pigs was unable to show any benefit from either treatment [7]. Also, studies in calves provide mixed results. One study investigating different navel dips found numerically fewer umbilical infections in two groups (chlorhexidine and dry nisin), than in the others (iodine and liquid nisin) [8], the difference was however insignificant, and the overall omphalitis rate was 9% (6/67 calves). These results contrast with another study in calves that found an omphalitis rate of zero within 24 h post-partum and no differences in the healing rate of the umbilical cord [9]. For human studies, the results are more convincing, and WHO [10] recommends antiseptic treatment of the umbilical cord, with chlorhexidine, in areas with a neonatal mortality rate above 30.^1^ One study in children found that disinfection within 24 h post-partum reduced mortality significantly compared to disinfection with chlorhexidine after 24 h [11]. Because of this, we wanted to test if disinfection with chlorhexidine followed by tying and cutting of the umbilical cord might influence mortality and UO prevalence in pigs.

The effect of non-steroidal anti-inflammatory drugs (NSAID) administration for newborn piglets on UO development has not been investigated, but there are studies investigating the effects of NSAID administration on newborn calves.

The anti-inflammatory effects of NSAIDs might be beneficial in reducing the prevalence of UO and may even reduce mortality.

A study from 2016 showed improved vigour and suckling reflex in newborn calves after meloxicam administration [12] and another found meloxicam improved average daily gain in calves from difficult births [13]. This agrees with another study that found meloxicam increased standing ability in calves with low vitality [14], and meloxicam seems not to have adverse effects on mortality [15].

We considered that part of the aetiology behind UO development is inflammation and infections, and therefore we wanted to assess the effect of disinfection and inflammatory drugs in combination expecting that the effects were synergistic instead of antagonistic.

The objective of this study was to examine the effect of different day 1 treatments on the development of UOs and mortality from birth to nursery unit.

The groups were: a control group receiving no treatment, an antibiotic group receiving amoxicillin, and an experimental group where the piglets had their umbilical cord disinfected with chlorhexidine, followed by tying and clipping, and lastly, they were injected with meloxicam. Additionally, other risk factors, previously shown to affect UO prevalence or mortality, such as sex, the status of the umbilical cord at inclusion, IUGR (intrauterine growth restriction), birth weight, individual antibiotic treatments, and cross fostering [7, 16–21] were examined for their effect on the prevalence of UOs and mortality.

## Results

The demographics of the 5674 included and individually ear-tagged pigs are shown in Table 1.

**Table 1:** Baseline demographics of 5674 included pigs grouped in three treatment groups

		Treatment group N (%)^a^			
Variable	Level	Control	Antibiotic	Experimental	N (%)
	Pigs total	1839 (32.4)	1923 (33.9)	1912 (33.7)	5674 (100)
	Loss to follow up	48 (26.7)	61 (33.9)	71 (39.4)	180 (3.2)
	Remaining pigs	1791 (32.6)	1862 (33.9)	1841 (33.5)	5494 (96.8)
Sex	Male	899 (32.3)	938 (33.7)	943 (33.9)	2780 (50.6)
	Female	892 (32.9)	923 (34.1)	895 (33.0)	2710 (49.3)
	NA^b^	0	1 (25)	3 (75)	4 (0.1)
Umbilical cord^c^	Newborn	276 (29.9)	307 (33.3)	340 (36.8)	923 (16.8)
	Starting to demarcate	282 (28.8)	303 (30.9)	395 (40.3)	980 (17.8)
	Clear demarcation	415 (32.2)	408 (31.6)	467 (36.2)	1290 (23.5)
	Damp only at base	817 (35.6)	843 (36.7)	634 (27.6)	2294 (41.8)
	NA	1 (14.3)	1 (14.3)	5 (71.4)	7 (0.1)
IUGR	Normal	1615 (32.1)	1696 (33.8)	1714 (34.1)	5025 (91.5)
	Mild	166 (38.1)	152 (34.9)	118 (27.1)	436 (7.9)
	Severe	9 (33.3)	11 (40.7)	7 (25.9)	27 (0.5)
	NA	1 (16.7)	3 (50)	2 (33.3)	6 (0.1)
Weight (kg)	Average Standard deviation	1.33 0.33	1.35 0.33	1.39 0.33	1.35 0.33
Individual antibiotic treatment^d^	Yes	78 (4.4)	63 (3.4)	70 (3.8)	211 (3.8)
	No	1713 (95.6)	1799 (96.6)	1771 (96.2)	5283 (96.1)
Cross fostering	Yes	447 (25.0)	449 (24.1)	408 (22.2)	1304 (23.7)
	No	1344 (75.0)	1413 (75.9)	1433 (77.8)	4190 (76.3)

^a^Number (%) of animals in each group

^b^NA = not available

^c^At inclusion, see Fig. 1 for more information

^d^In the farrowing unit

The piglets weighed at inclusion on average 1.35 kg with a standard deviation of 0.33 kg. Male piglets made up 50.6% of the total population (2780/5494), while female piglets made up 49.3% (2710/5494). A total of 7.9% (436/5494) were classified as having mild IUGR, and 0.5% (27/5494) as having severe IUGR. Individual antibiotic treatments were recorded for 3.8% (211/5494) of the piglets in the farrowing unit, and a total of 23.7% (1304/5494) of the piglets were cross fostered (Table 1).

Piglets included per week batch varied from 404 to 957, except for the final batch where only 63 pigs were included to reach the desired sample size.

The clinical examination was conducted in the nursery unit six weeks after weaning, where 79.7% (4378/5494) of the included pigs were examined. The examination age ranged from 57 to 93 days, but 83.4% (3653/4378) of the pigs were between 63 and 65 days old. The pigs were on average 66.4 days old at the examination, with a median of 65 days. The UO prevalence was 4.3% at the clinical examination (Table 2).

**Table 2:** The distribution of 5494 remaining pigs across treatment groups and the outcomes umbilical outpouching (UO) or dead

		Treatment group N (%)^a^			N(%)
		Control	Antibiotic	Experimental	Total
Distribution across groups	Total	1791 (32.6)	1862 (33.9)	1841 (33.5)	5494 (100)
**Dead before clinical examination**	**Total**	**381** **(21.3)**	**365** **(19.6)**	**370** **(20.1)**	**1116** **(20.3)**
Dead with UO	Yes	9 (2.4)	12 (3.3)	7 (1.9)	28 (2.5)
	No	372 (97.6)	353 (96.7)	363 (98.1)	1088 97.5)
**Examined 6 weeks after weaning**	**Total**	**1410** **(78.7)**	**1497** **(80.4)**	**1471** **(79.9)**	**4378** **(79.7)**
UO 6 weeks after weaning	Yes	58 (4.1)	65 (4.3)	65 (4.4)	188 (4.3)
	No	1352 (95.9)	1432 (95.7)	1406 (95.6)	4190 (95.7)
**Dead in nursery unit after clinical examination**	**Total**	**30** **(1.7)**	**26** **(1.4)**	**24** **(1.3)**	**80** **(1.5)**
Dead with UO after clinical examination	Yes	20 (66.6)	18 (69.2)	17 (70.8)	55 (68.8)
	No	10 (33.3)	8 (30.8)	7 (29.2)	25 (31.2)
**Overall UO prevalence**^**b**^		69 (3.9)	78 (4.2)	74 (4.0)	**221** **(4.0)**
**Overall mortality**^**c**^		411 (22.9)	391 (21)	394 (21.4)	**1196** **(21.8)**

^a^Number (%) of animals in each group

^b^Including UO found after initial clinical examination

^c^Including deaths occurring after clinical examination

The multivariable logistic regression model showed no statistically significant differences in UO prevalence between the different treatment groups (*p* = 0.85 Table 3).

**Table 3:** Logistic regression model for the multivariable analysis of the outcome umbilical outpouching (UO) yes/no

Variable	Level	Estimate	OR (95% CI)^a^	P
Group	Control	0	1	0.85
	Antibiotic	0.10	1.10 (0.79–1.53)	
	Experimental	0.04	1.04 (0.74–1.46)	
*Sex*	Male	0	1	< 0.001
	Female	0.53	1.70 (1.29–2.24)	
Random effect		Variance	Standard deviation	
Week batch		0.01149	0.1072	

^a^OR = odds ratio, CI = confidence interval

The variable IUGR was removed from the univariate analysis for UO prevalence because of a very large impact on mortality: 51% (221/436) of the pigs with mild IUGR died and 96% (26/27) of the pigs with severe IUGR, 92% (209/227) of the IUGR pigs died within 15 days of age. In other words, the IUGR pigs never had a chance to develop UOs and the data makes IUGR seem falsely protective of UOs.

Results from the univariate analysis for UO prevalence can be seen in Additional file 1: Table S1.

The only variable, from the univariate analysis, with a significant effect on UO prevalence was sex (*p* < 0.001).

Table 3 shows the result of the final model after assessing confounding and possible interactions. The treatment group was forced into the multivariable analysis as the primary variable of interest, despite being insignificant. Sex was the only variable found significant for the prevalence of UOs, with females having an OR of 1.7 (CI 1.28–2.25) for developing UOs compared to male pigs. No relevant 2-way interactions were identified, and no variables confounded the significant variables.

The overall mortality was 21.8% (1196/5494) from birth until departure from the nursery unit, and 93.3% (1116/1196) of the dead pigs died before the clinical examination at week 6 after weaning. The mean age of death was 14.6 days, with a median of 4 days. Mortality before 15 days of age was 15.7% (864/5494). UOs were recorded in 2.5% (28/1116) of the pigs dying before the clinical examination and in 68.8% (55/80) of the pigs dying after the clinical examination (Table 2).

The multivariable logistic regression model showed no statistically significant differences in mortality between the three treatment groups (*p* = 0.33 Table 4).

**Table 4:** Logistic regression model for the multivariable analysis of the outcome dead yes/no

Variable	Level	Estimate	OR (95% CI)	P
Group	Control	0	1	0.33
	Antibiotic	-0.08	0.92 (0.78–1.09)	
	Experimental	0.04	1.04 (0.88–1.23)	
Sex	Male	0	1	< 0.001
	Female	-0.27	0.76 (0.66–0.87)	
IUGR	Normal	0	1	< 0.001
	Mild	0.54	1.7 (1.34–2.18)	
	Severe	3.80	44.76 (6.03–332.06)	
Weight quartile	Fourth > 1.6 kg	0	1	< 0.001
	Third 1.36–1.59 kg	0.29	1.33 (1.05–1.68)	
	Second 1.11–1.35 kg	0.75	2.13 (1.73–2.61)	
	First < 1.11 kg	1.60	4.96 (3.94–6.24)	
Cross fostering	No	0	1	0.004
	Yes	0.23	1.26 (1.07–1.46)	
Random effect		Variance	Standard deviation	
Week batch		0.01379	0.1174	

Results from the univariate analysis for mortality can be studied in Additional file 2: Table S2.

The variables sex, IUGR, weight quartile, and cross fostering were all significant in the univariate analysis.

The treatment group was forced into the multivariable analysis, as the primary variable of interest, despite being insignificant. In the final model for death (yes/no), sex (*p* < 0.001), IUGR (*p* < 0.001), weight (*p* < 0.001), and cross fostering (*p* = 0.004) had a significant influence on mortality. Female pigs had lower odds of dying (OR 0.76) compared to male pigs. Pigs classified as mild IUGR had increased odds of dying (OR 1.7) and if they were classified as severe IUGR OR was 44.8. Cross fostered pigs also had an increased risk of dying (OR 1.3) (Table 4).

## Discussion

The prevalence of UOs did not differ significantly between the three treatment groups, which agrees with other studies [3, 4, 22] showing insignificant effects of antibiotics but disagrees with others [5, 23]. Neither the treatment with NSAID, disinfection, cutting, and tying of the umbilical cord nor the treatment with antibiotics was superior to no treatment at all.

The only variable affecting the prevalence of UOs was sex, with female pigs having higher OR, which is also seen in other studies [7, 16]. Reasons for this are so far unknown, but one can hypothesize that hereditary elements such as growth and sex hormones may contribute to the healing of the umbilical plate and increase the risk of UO in female pigs.

In contrast to another Danish study [7] individual treatments did not affect the prevalence of UOs. It is noteworthy that this herd had a low level of individual treatments, which may be partly responsible for the very high mortality seen in this herd and reduce power for the variable.

This study could not show that IUGR increased the risk of UO prevalence simply because the IUGR piglets died before they developed UOs.

This study found no significant differences in mortality among the three groups. This agrees with an American study, which found no differences in mortality nor abdominal herniation, from birth until weaning, between three groups treated within 24 h postpartum. The groups were a negative control, injection with penicillin, or injection with ceftiofur [22]. It does however contrast with a Finnish study that reduced mortality and prevalence of umbilical hernias significantly in a group receiving amoxicillin one day after birth, compared to a negative control group [5]. It can be speculated whether the Finnish findings were the result of a de novo effect of introducing antibiotics. The herd in this study already used metaphylactic antibiotics and antibiotic resistance might therefore reduce the effects measured.

Sex, IUGR, birth weight, and cross fostering were all factors significantly influencing the OR of death. Males have previously been shown to have a higher risk of mortality [18] as well as IUGR piglets have higher risk [17], pigs of low birth weight are at higher risk [20, 21] and cross fostered piglets are at a higher risk of dying [19, 24].

Although a minor reduction may exist, the sample size was calculated to find a reduction of at least 40%. A potential reduction should be big enough to justify the workload required for disinfecting, cutting, and tying all umbilical cords in all piglets. Only disinfecting the cord is less labour intensive and might be worth considering.

Cords were tied in this study because they were cut when still wet, and we wanted to prevent bleeding and having an open umbilical cord, this procedure might however affect healing/ increase the risk of infection. To the author's knowledge, there are no studies examining this, however since the cords detach early in pigs, the authors consider this a minor problem.

Tying of the umbilical cord is recommended in humans, but in multiparous animals, such as pigs, the umbilical cord is thinner and detaches earlier (2–3 days) than in uniparous species, and cord tying might therefore be irrelevant in pigs.

Adding a later disinfection of the umbilical cord e.g., at castration/ iron injection/ coccidiosis treatment, might help prevent ascending infection through the healing umbilicus, especially for pigs that are kept in a much dirtier environment than babies.

The drug of choice for disinfection should probably be chlorhexidine [8] since iodine is neutralized in the presence of organic material and might work poorly on newborn piglets covered in mucous and foetal membranes.

Previous studies in calves gave no reason to suspect that the administration of meloxicam would be detrimental to newborn piglets, however, it is possible that meloxicam could have had some adverse effects and thus ruined possible positive effects of the disinfection. It would be preferable to isolate the NSAID, disinfection, and cutting and tying from each other if the study were to be repeated.

The nature of the treatments prevented blinding of the study; however, the umbilical cord and suture disappeared a long time before outcome measurement (6 weeks after weaning), and the researcher examining the pigs was unable to link individual pigs to treatment groups.

For practical reasons, it was decided to examine all pigs in the weaner section that were to be sold next. That way pigs were as old as possible at examination. Most of the pigs were then nine weeks old at the examination. The smallest pigs weaned from every week batch, however, were placed in baby units for 2 weeks, and were subsequently mixed with the week batch weaned two weeks later, thus being 11 weeks old at the examination (see Additional file 3: Fig. S1 for illustrations). Upon inspection, however, the pigs were, approximately the same physical size within the same unit.

If the pigs had been examined again later, we might have found more pigs with UOs. A total of 80 pigs died or were euthanized in the nursery after clinical examination at 6 weeks of age, and 5 of these had developed large outpouchings despite normal findings at the clinical examination (see Additional file 4: Fig. S2). It is known from earlier studies that some UOs disappear, and some occur at a later stage [7, 16]. In our study there is some indication that age affects the prevalence of UO (data not shown), however, it cannot strictly be concluded that the risk of UO prevalence increases with age, it could also be that pigs examined at a later age, were delayed (e.g. reduced growth) through the system because of the UO.

## Conclusions

This study showed that the prevalence of UO and mortality from birth to nursery unit. was not reduced following the administration of amoxicillin or meloxicam in combination with disinfection and tying of the umbilical cord.

Sex was the only variable affecting UO prevalence in this study, with females being at higher risk. The variables sex, IUGR, birth weight, and cross fostering all affected mortality. Males had higher odds of dying, as well as piglets recorded with some degree of IUGR. Also, low birth weight increased the odds of dying for all weight quartiles compared to the heaviest piglets, as well as cross fostering negatively affected the odds ratio of dying.

## Methods

### Study design

The study was a randomised controlled clinical field trial with three groups of piglets receiving different treatments at birth. The outcomes were the prevalence of UOs and the mortality from birth until leaving the nursery unit, starting 7 weeks after weaning. The prevalence of UOs was measured six weeks after weaning or at death if the pig died before departure from the nursery.

### Sample size

Assuming a prevalence of 4% UOs in the group of control pigs [1] and to detect a reduction of 1.6 percentage points between the control group and the two other groups, the required sample size for each group was 1496 pigs using an equation to detect differences between proportions [25]. To account for missing or dead pigs the final sample size was increased by 20% to 1870 pigs in each group.

### Study-herd and selection of pigs

The study-herd was selected by convenience in the eastern part of Denmark. The herd was an indoor herd with 1800 sows. According to the Danish Specific Pathogen Free (SPF) declaration the herd was infected with *Mycoplasma hyopneumoniae* but free from Actinobacillus pleuropneumoniae, PRRS, Brachyspira hyodysenteriae, Pasteurella multocida, Sarcoptes scabiei var suis, and Haematopinus suis [26].

The piglets were crossbreds between Landrace x Yorkshire x Duroc. Sows were kept in crates in the farrowing pen with partially solid and slatted floors. In the corner of each crate was a piglet nest with a heating lamp and a cover.

The inclusion of pigs took part from November 2021 until January 2022, and the study finished in May 2022. Piglets were included in the study from Sunday to Tuesday each week, from nine different batches of farrowing sows.

**Fig. 1:**
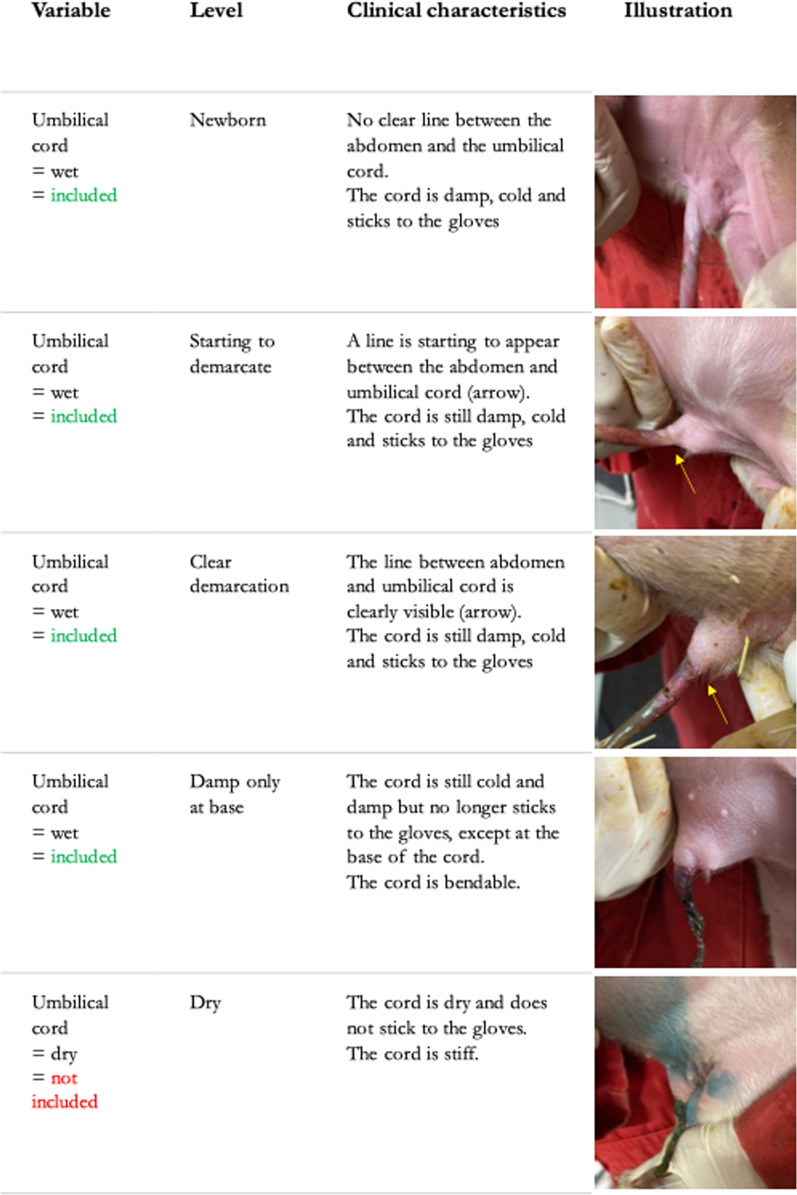
Classification of umbilical cords of piglets at inclusion. The demarcation line (yellow arrows) is where the cord detaches from the abdomen at 2–3 days of age

The inclusion of piglets started each morning. Inclusion criteria were wet umbilical cords, according to Fig. 1. Piglets were included from sows in farrow to ensure that as many piglets as possible still had wet umbilical cords. The status of the sow was not relevant for the inclusion of the piglets.

Exclusion criteria were cold/non-moving piglets, evident malformations, lameness/joint swellings, neurological symptoms, reduced general condition, dyspnoea, hypothermia, or dry umbilical cords according to Fig. 1. The pigs were part of the study until death or selling from the nursery unit starting seven weeks after weaning.

### Study procedures

At inclusion, all pigs had an electronic ear tag placed in the left ear, followed by weighing in a weighing wagon (Bjerringbro Vægte APS). Sex, umbilical cord categorization following Fig. 1, and an evaluation of intrauterine growth restriction (IUGR) based on definitions by Bahnsen et al. (2021)[27] were noted. The piglets were randomly allocated to one of three treatment groups by throwing a dice. All evaluations and treatments at birth were performed by the first author assisted by student helpers.

The three treatment groups were:

- Control (CO): Received no treatment.
- Antibiotic (AB): Intramuscular injection with 0.5 ml amoxicillin (*Vetrimoxin® LA 150 mg/ ml).*
- Experimental (EXP): Disinfection of the navel cord using 5% aqueous chlorhexidine and a spray bottle, intramuscular injection of 0.15 ml of 5% meloxicam (Metacam 5% meloxicam solution for injection in cattle and pigs), tying of the navel cord with silk suture USP 1 one cm below the abdomen and cutting of the cord using scissors two cm below the suture.

Antibiotics or NSAIDs were injected intramuscularly behind the left ear using automatic syringes. New needles were applied every morning and for every 50 pigs included in the study.

After inclusion, the pigs followed the normal procedures of the farm, with the exemption that prophylactic or metaphylactic antibiotic treatment was not allowed in piglets before weaning. The farm staff was responsible for cross fostering (including the decision of which piglets to cross foster) and recordings. The farm staff was also responsible for the health and welfare of the animals, and individual treatments with antibiotics according to the farm’s standard operational procedures and veterinary instructions were allowed. Individual treatments were recorded in the farrowing unit but not in the nursery unit. The piglets were weaned Friday in the third week after birth, so most of the pigs were 24–26 days old at weaning. After weaning piglets were moved to another facility. The nursery pens consisted of a covered resting area with a solid floor, an activity area with a slatted floor, and had climate control system. The weaners were fed wet feed restrictively.

### Clinical and postmortem examinations

The pigs were checked for UOs 6 weeks after weaning. The pigs were inspected following the procedures and definitions introduced by Hansen et al. (2024) for the assessment of weaners [1] using the same cut-off of 2 × 2 cm (height x width) to record clinically relevant UOs. Trained technicians screened all weaners by palpating the abdominal area and then spray-marked pigs with abnormalities, bulges, or uncertainties. Marked pigs were then fixated with a herding board and examined standing by the first author [1].

All pigs dying before departure from the nursery unit were examined by the first author at the herd. Examination focused on the umbilical area and the presence/ absence of UOs. The examinations were not performed to establish a cause of death.

### Statistical analysis

The individual pig was the experimental unit for all analyses. All data were analysed, and graphs were made, in Rstudio [28] using functions from the Tidyverse package [29].

Logistic regression models were built using the lme4 package in R [30]. Univariate logistic regression analyses were used to screen the variables and the following variables were screened with the outcome UO (yes/ no) and the outcome dead yes/no: Treatment group (control, antibiotic, experimental), sex (male, female), umbilical cord status (newborn, starting to demarcate, clear demarcation, only damp at base), weight quartile (1st, 2nd, 3rd, and 4th), individual antibiotic treatments (yes, no), and cross fostering (yes, no). Weight quartiles were derived based on the median (included in the 2nd quartile), and 25 & 75% quartiles.

All variables with *p* values < 0.25 in the univariate logistic regression analyses were included in the multivariable logistic regression analyses. Before the analysis the individual risk factors were assessed for association between each other using cross-tabulation and chi-sq, and for correlation using Spearman’s correlation for ordered data. Confounding was further assessed by adding excluded insignificant variables to the model and keeping those that resulted in more than 20% change of estimates of significant variables. Relevant two-way interaction terms were added to the model, and none were significant. The drop1 function in R was applied using backward elimination until the final model only included variables with *p* values < 0.05. All analyses included the week batch as a random effect.

## Supplementary Information

**Additional file 1: Table S1**. Univariate logistic regression analysis - outcome UO yes/no. Table showing the results from the univariate analysis of the outcome UO yes/ no. Significant variables and variables with tendencies are marked with italics

**Additional file 2: Table S2**. Univariate logistic regression analysis - outcome dead yes/ no. Table showing the results from the univariate analysis of the outcome UO yes/ no. Significant variables and variables with tendencies are marked with italics

**Additional file 3: Fig. S1**. Age distribution of pigs examined for umbilical outpouchings grouped by treatment group. Figure S1 shows the age distribution of the examined pigs along with an explanation

**Additional file 4: Fig. S2**. Piglet´s age at death grouped by cause of death. Figure 3 shows how old the pigs were when they died, and why they died

## Data Availability

The datasets used and/or analysed during the current study are available from the corresponding author upon reasonable request.

## Abbreviations

IUGR: Intrauterine growth restriction
NSAID: Nonsteroidal anti-inflammatory drugs
UO: Umbilical outpouching

## Footnotes

1 30 deaths/ 1000 liveborn within 28 days post-partum.
