# Correction: Prevention of umbilical outpouchings and mortality in pigs: Meloxicam, tying, cutting, and chlorhexidine versus amoxicillin or no treatment? A clinical field trial

**DOI:** 10.1186/s40813-025-00443-8

**Published:** 2025-06-02

**Authors:** Marie-Louise Hansen, Inge Larsen, Tina Birk Jensen, Charlotte Sonne Kristensen, Ken Steen Pedersen

**Affiliations:** 1 Department of Veterinary and Animal Sciences, Faculty of Health and Medical Sciences, University of Copenhagen, Grønnegårdsvej 2, Frederiksberg C, 1870 Denmark; 2 SEGES Innovation, Agro Food Park 15, Aarhus, 8200 Denmark; 3 Ø-Vet A/S, Køberupvej 33, Næstved, 4700 Denmark

**Correction: Porc Health Manag 10**,** 10 (2024).**

10.1186/s40813-024-00358-w

Following publication of the original article [1], the authors reported an error in the references section.

It was published as follows:

1. Hansen M-L, Jensen TB, Kristensen CS, Larsen I, Pedersen KS. Umbilical outpouchings in Danish piglets and weaners: prevalence and clinical characteristics—a cross-sectional study at herd level. Porcine Health Manag. 2024;10:3. 10.1186/s40813-023-00352-8.
2. Danish Agriculture and Food Council. STATISTICS 2022 Pigmeat [Internet]0.2023. Available from: https://lf.dk/tal-og-analyser/statistik/svin/statistik-svin/statistik-gris-2022.
3. Searcy-Bernal R, Gardner IA. Effects of and factors associated with umbilical hernias in a swine herd. J Am Vet Med Assoc. 1994;204:1660–4.
4. Rutten-Ramos SC, Deen J. Association between umbilical hernias and genetic line in a swine multiplication herd and methods to differentiate the role of sire in the incidence of umbilical hernias in offspring. J Swine Health Prod. 2006;14:317–22.
5. Yun J, Olkkola S, Hänninen M-L, Oliviero C, Heinonen M. The effects of amoxicillin treatment of newborn piglets on the prevalence of hernias and abscesses, growth and ampicillin resistance of intestinal coliform bacteria in weaned pigs. PLoS ONE. 2017;12(2):e0172150.
6. Robinson AL, Colpoys JD, Robinson GD, Hines EA, Timms LL, Edwards EM, et al. The effect of antiseptic compounds on umbilical cord healing in piglets in a commercial facility. J Swine Health Prod. 2016;24:212–5.
7. Larsen I, Baekbo P, Nielsen JP. Umbilical Outpouchings in two Danish herds raising pigs with minimal use of antibiotics-Results from a field trial evaluating the efficacy of autogenous vaccines and iodine application. Prev Vet Med. 2023;214:105905. 10.1016/j.prevetmed. 2023.105905.
8. Fordyce AL, Timms LL, Stalder KJ, Tyler HD. Short communication: The effect of novel antiseptic compounds on umbilical cord healing and incidence of infection in dairy calves. J Dairy Sci. 2018;101:5444–8.
9. Robinson AL, Timms LL, Stalder KJ, Tyler HD. The effect of 4 antiseptic compounds on umbilical cord healing and infection rates in the first 24 h in dairy calves from a commercial herd. J Dairy Sci. 2015;98:5726–8. 10.3168/jds.2014-9235.
10. World Health Organisation. WHO recommendations on maternal and newborn core for a positive postnatal experience [Internet]. 2022. Available from: https://www.who.int/publications/i/item/9789240044074.
11. Mullany LC, Darmstadt GL, Khatry SK, Katz J, Leclerq SC, Shrestha S, et al. Topical applications of chlorhexidine to the umbilical cord for prevention of omphalitis and neonatal mortality in southern Nepal: a community based, cluster-randomised trial. Lancet. 2006;367:910–8.
12. Murray CF, Dufeld TF, Haley DB, Pearl DL, Veira DM. The effect of meloxicam NSAID therapy on the change in vigor, suckling reflex, blood gas measures, milk intake and other variables in newborn dairy calves. J Vet Sci Anim Husb. 2016;4:103.
13. Pearson JM, Pajor EA, Campbell JR, Caulkett NA, Levy M, Dorin C, et al. Clinical impacts of administering a nonsteroidal anti-inflammatory drug to beef calves after assisted calving on pain and inflammation, passive immunity, health, and growth. J Anim Sci. 2019;97:1996–2008.
14. Kovács L, Kézér F, Ruf F, Samardzija M, Szenci,. Single -dose meloxicam treatment improves standing ability of low -vitality dairy calves. J Dairy Sci. 2022;105:1618–24.
15. Pearson JM, Pajor E, Campbell J, Levy M, Caulkett N, Windeyer MC. A randomised controlled trial investigating the effects of administering a non -steroidal anti -inflammatory drug to beef calves assisted at birth and risk factors associated with passive immunity, health, and growth. Vet Rec Open. 2019;6:e0003646.
16. Hovmand -Hansen T, Jensen TB, Vestergaard K, Nielsen MBF, Leifsson PS, Jensen HE. Early risk factors, development, disappearance and contents of umbilical outpouching in Danish pigs. Livest Sci. 2021;251: 104,654.
17. Hansen CF, Hales J, Amdi C, Moustsen VA. Intrauterine growth -restricted piglets defined by their head shape have impaired survival and growth during the suckling period. Anim Prod Sci. 2019;59:1056–62.
18. Baxter EM, Jarvis S, Palarea-Albaladejo J, Edwards SA. The weaker sex? The propensity for male -biased piglet mortality. PLoS ONE. 2012;7(1):e30318.
19. Nielsen CL, Krogh MA, Sørensen JT, Kongsted H. A field trial on the effect of cross -fostering and weaning age on daily gain and disease resilience in weaned pigs. Prev Vet Med. 2022;208:105762.
20. Szczotka A, Hayman K, Ratlif B, Rademacher C, Swalla R, Hoogland M, et al. Effects of antibiotic and non -antibiotic interventions applied to swine umbilici within the first 24 h of life on the incidence of umbilical infection, growth, and mortality. In: 50th Annual Meeting of the American Association of Swine Veterinarians. Orlando; 2019. p. 94–5.
21. Reiman J, Greiner L, Lowe J, Connor J. Bacterial infection of the navel cord in neonatal piglets: A follow up on prevalence and clinical outcomes. In: AASV Annual Meeting: Integrating Science, Welfare, and Economics in Practice. 2012. p 55–6.
22. Houe H, Kjær Ersbøl A, Toft N. Introduction to veterinary epidemiology [Internet]. 1st. ed. Frederiksberg C: Biofolia; 2004 [cited 2021 Sep 6]. Avail able from: https://www.cabdirect.org/cabdirect/abstract/20053100612.
23. Danish Agriculture and Food Council. Specific Pathogen Free pigs SPF [Internet]. 1971 [cited 2023 Sep 22]. Available from: https://spfsus.dk/en.
24. Bahnsen I, Riddersholm KV, De Knegt LV, Bruun TS, Amdi C. The effect of different feeding systems on salivary cortisol levels during gestation in sows on herd level. Animals. 2021. 10.3390/ani11041074.
25. Posit Team PSP. R Studio: Integrated Development Environment for R [Internet]. Boston, MA; 2023 [cited 2023 Jun 23]. Available from: http://www.posit.co/.
26. Wickham H, Averick M, Bryan J, Chang W, McGowan L, François R, et al. Welcome to the Tidyverse. J Open Source Softw. 2019;4:1686.
27. Bates D, Mächler M, Bolker B, Walker S. Fitting linear mixed -effects models using lme4. J Stat Softw. 2015;67:1–48.

It should be as follows:

1. Hansen M-L, Jensen TB, Kristensen CS, Larsen I, Pedersen KS. Umbilical outpouchings in Danish piglets and weaners: prevalence and clinical characteristics—a cross-sectional study at herd level. Porcine Health Manag. 2024;10:3.
2. Danish Agriculture and Food Council. STATISTICS 2022 Pigmeat [Internet]. 2023. Available from: https://lf.dk/tal-og-analyser/statistik/svin/statistik-svin/statistik-gris-2022.
3. Searcy-Bernal R, Gardner IA. Effects of and factors associated with umbilical hernias in a swine herd. J Am Vet Med Assoc. 1994;204:1660–4.
4. Rutten-Ramos SC, Deen J. Association between umbilical hernias and genetic line in a swine multiplication herd and methods to differentiate the role of sire in the incidence of umbilical hernias in offspring. Journal of Swine Health and Production. 2006;14:317–22.
5. Yun J, Olkkola S, Hänninen M-L, Oliviero C, Heinonen M. The effects of amoxicillin treatment of newborn piglets on the prevalence of hernias and abscesses, growth and ampicillin resistance of intestinal coliform bacteria in weaned pigs. PLoS One. 2017;12.
6. Robinson AL, Colpoys JD, Robinson GD, Hines EA, Timms LL, Edwards EM, et al. The effect of antiseptic compounds on umbilical cord healing in piglets in a commercial facility. J Swine Health Prod. 2016;24:212–5.
7. Larsen I, Baekbo P, Nielsen JP. Umbilical Outpouchings in two Danish herds raising pigs with minimal use of antibiotics-Results from a field trial evaluating the efficacy of autogenous vaccines and iodine application. Prev Vet Med. 2023;214:105905.
8. Fordyce AL, Timms LL, Stalder KJ, Tyler HD. Short communication: The effect of novel antiseptic compounds on umbilical cord healing and incidence of infection in dairy calves. J Dairy Sci. 2018;101:5444–8.
9. Robinson AL, Timms LL, Stalder KJ, Tyler HD. The effect of 4 antiseptic compounds on umbilical cord healing and infection rates in the first 24 h in dairy calves from a commercial herd. Journal of Dairy Science. 2015;98:5726–8.
10. World Health Organisation. WHO recommendations on maternal and newborn core for a positive postnatal experience [Internet]. 2022. Available from: https://www.who.int/publications/i/item/9789240044074.
11. Mullany LC, Darmstadt GL, Khatry SK, Katz J, Leclerq SC, Shrestha S, et al. Topical applications of chlorhexidine to the umbilical cord for prevention of omphalitis and neonatal mortality in southern Nepal: a community-based, cluster-randomised trial. Lancet. 2006;367:910–8.
12. Murray CF, Duffield TF, Haley DB, Pearl DL, Veira DM. The Effect of Meloxicam NSAID Therapy on the Change in Vigor, Suckling Reflex, Blood Gas Measures, Milk Intake and Other Variables in Newborn Dairy Calves. J Vet Sci Anim Husb. 2016;4:103.
13. Pearson JM, Pajor EA, Campbell JR, Caulkett NA, Levy M, Dorin C, et al. Clinical impacts of administering a nonsteroidal anti-inflammatory drug to beef calves after assisted calving on pain and inflammation, passive immunity, health, and growth. J Anim Sci. 2019;97:1996–2008.
14. Kovács L, Kézér F, Ruff F, Samardzija M, Szenci O. Single-dose meloxicam treatment improves standing ability of low-vitality dairy calves. J Dairy Sci. 2022;105:1618–24.
15. Pearson JM, Pajor E, Campbell J, Levy M, Caulkett N, Windeyer MC. A randomised controlled trial investigating the effects of administering a non-steroidal anti-inflammatory drug to beef calves assisted at birth and risk factors associated with passive immunity, health, and growth. Vet Rec Open. 2019;6.
16. Hovmand-Hansen T, Jensen TB, Vestergaard K, Nielsen MBF, Leifsson PS, Jensen HE. Early risk factors, development, disappearance and contents of umbilical outpouching in Danish pigs. Livest Sci. 2021;251:104654.
17. Hansen CF, Hales J, Amdi C, Moustsen VA. Intrauterine growth-restricted piglets defined by their head shape have impaired survival and growth during the suckling period. Anim Prod Sci. 2019;59:1056–62.
18. Baxter EM, Jarvis S, Palarea-Albaladejo J, Edwards SA. The weaker sex? the propensity for male-biased piglet mortality. PLoS One. 2012;7.
19. Nielsen CL, Krogh MA, Sørensen JT, Kongsted H. A field trial on the effect of cross-fostering and weaning age on daily gain and disease resilience in weaned pigs. Prev Vet Med. 2022;208.
20. Ferrari C V., Sbardella PE, Bernardi ML, Coutinho ML, Vaz IS, Wentz I, et al. Effect of birth weight and colostrum intake on mortality and performance of piglets after cross-fostering in sows of different parities. Prev Vet Med. 2014;114:259–66.
21. Fix JS, Cassady JP, Holl JW, Herring WO, Culbertson MS, See MT. Effect of piglet birth weight on survival and quality of commercial market swine. Livest Sci. 2010;132:98–106.
22. Szczotka A, Hayman K, Ratliff B, Rademacher C, Swalla R, Hoogland M, et al. Effects of antibiotic and non-antibiotic interventions applied to swine umbilici within the first 24 h of life on the incidence of umbilical infection, growth, and mortality. 50th Annual Meeting of the American Association of Swine Veterinarians. Orlando; 2019. p. 94–5.
23. Reiman J, Greiner L, Lowe J, Connor J. Bacterial infection of the navel cord in neonatal piglets: A follow up on prevalence and clinical outcomes. AASV Annual Meeting: Integrating Science, Welfare, and Economics in Practice. 2012. p. 55–6.
24. Díaz JAC, Manzanilla EG, Diana A, Boyle LA. Cross-fostering implications for pig mortality, welfare and performance. Front Vet Sci. 2018;5.
25. Houe H, Ersbøll AK, Toft N. Introduction to veterinary epidemiology. Copenhagen: Biofolia; 2004.
26. Danish Agriculture and Food Council. Specific Pathogen Free pigs SPF. 1971.
27. Bahnsen I, Riddersholm K V, De Knegt L V, Bruun TS, Amdi C. The Effect of Different Feeding Systems on Salivary Cortisol Levels during Gestation in Sows on Herd Level. Animals. 2021;
28. Posit Team PSP. R Studio: Integrated Development Environment for R. Boston, MA; 2023.
29. Wickham H, Averick M, Bryan J, Chang W, McGowan L, François R, et al. Welcome to the Tidyverse. J Open Source Softw. 2019;4:1686.
30. Bates D, Mächler M, Bolker B, Walker S. Fitting Linear Mixed-Effects Models Using **lme4**. J Stat Softw. 2015;67:1–48.

The original article [1] has been updated.
