# Supplementary material for: Prevention of umbilical outpouchings and mortality in pigs: Meloxicam, tying, cutting, and chlorhexidine versus amoxicillin or no treatment? A clinical field trial
Source: Porcine Health Manag. 2024 Feb 16;10:10. doi: 10.1186/s40813-024-00358-w (PMC10874036; doi:10.1186/s40813-024-00358-w)
Supplement: Supplementary file 1 — Additional file 1: Table S1. Univariate logistic regression analysis - outcome UO yes/no. Table showing the results from the univariate analysis of the outcome UO yes/ no. Significant variables and variables with tendencies are marked with italics [file 40813_2024_358_MOESM1_ESM.docx]

Table S1 Univariate logistic regression analysis - outcome UO yes/no^[[1]](#footnote-1)^

| **Variable** | **Level** | **Estimate** | **OR (95% CI)** | **P** |
| --- | --- | --- | --- | --- |
| Group | Control | 0 | 1 | 0.86 |
|  | Antibiotic | 0.09 | 1.09 (0.79-1.53) |  |
|  | Experimental | 0.03 | 1.04 (0.74-1.45) |  |
| *Sex* | Male | 0 | 1 | <0.001 |
|  | Female | 0.53 | 1.70 (1.29-2.24) |  |
| Umbilical cord | Newborn | 0 | 1 | 0.84 |
|  | Starting to demarcate | -0.13 | 0.87 (0.56-1.37) |  |
|  | Clear demarcation | -0.20 | 0.82 (0.53-1.26) |  |
|  | Damp only at base | -0.14 | 0.87 (0.59-1.28) |  |
| Weight quartile | Fourth > 1.6 kg | 0 | 1 | 0.71 |
|  | Third 1.36-1.59 kg | -0.20 | 0.82 (0.56-1.22) |  |
|  | Second 1.11-1.35 kg | -0.10 | 0.91 (0.64-1.28) |  |
|  | First < 1.11 kg | -0.20 | 0.81 (0.52-1.22) |  |
| Individual AB treatment | No | 0 | 1 | 0.24 |
|  | Yes | 0.38 | 1.46 (0.80-2.67) |  |
| Cross fostering | No | 0 | 1 | 0.29 |
|  | Yes | 0.17 | 1.18 (0.87-1.60) |  |

Significant variables and variables with tendencies are marked with *italics.*

1. Week batch included as random effekt [↑](#footnote-ref-1)
