# Supplementary material for: Prevention of umbilical outpouchings and mortality in pigs: Meloxicam, tying, cutting, and chlorhexidine versus amoxicillin or no treatment? A clinical field trial
Source: Porcine Health Manag. 2024 Feb 16;10:10. doi: 10.1186/s40813-024-00358-w (PMC10874036; doi:10.1186/s40813-024-00358-w)
Supplement: Supplementary file 2 — Additional file 2: Table S2. Univariate logistic regression analysis - outcome dead yes/ no. Table showing the results from the univariate analysis of the outcome UO yes/ no. Significant variables and variables with tendencies are marked with italics [file 40813_2024_358_MOESM2_ESM.docx]

Table S2 Univariate logistic regression analysis - outcome dead yes/ no^[[1]](#footnote-1)^

| **Variable** | **Level** | **Estimate** | **OR (95% CI)** | **P** |
| --- | --- | --- | --- | --- |
| Group | Control | 0 | 1 | 0.32 |
|  | Antibiotic | -0.11 | 0.89 (0.76-1.04) |  |
|  | Experimental | -0.08 | 0.92 (0.79-1.08) |  |
| *Sex* | Male | 0 | 1 | 0.0077 |
|  | Female | -0.18 | 0.84 (0.74-0.95) |  |
| Umbilical cord | New-born | 0 | 1 | 0.81 |
|  | Starting to demarcate | -0.06 | 0.94 (0.76-1.18) |  |
|  | Clear demarcation | 0.04 | 1.04 (0.85-1.28) |  |
|  | Damp only at base | 0.00 | 1.00 (0.83-1.21) |  |
| *Intrauterine growth restriction* | Normal | 0 | 1 | < 0.001 |
|  | Mild | 1.50 | 4.48 (3.65-5.49) |  |
|  | Severe | 4.76 | 116.61 (16.38-830.09) |  |
| *Weight quartile* | Fourth > 1.6 kg | 0 | 1 | < 0.001 |
|  | Third 1.36-1.59 kg | 0.27 | 1.32 (1.04-1.66) |  |
|  | Second 1.11-1.35 kg | 0.75 | 2.11 (1.72-2.60) |  |
|  | First < 1.11 kg | 1.87 | 6.51 (5.30-8.00) |  |
| Individual AB treatment | No | 0 | 1 | 0.81 |
|  | Yes | 0.04 | 1.04 (0.75-1.45) |  |
| *Cross fostering* | No | 0 | 1 | < 0.001 |
|  | Yes | 0.53 | 1.71 (1.48-1.97) |  |

Significant variables and variables with tendencies are marked with *italics.*

1. Week batch included as random effect [↑](#footnote-ref-1)
