# Supplementary material for: Prevention of umbilical outpouchings and mortality in pigs: Meloxicam, tying, cutting, and chlorhexidine versus amoxicillin or no treatment? A clinical field trial
Source: Porcine Health Manag. 2024 Feb 16;10:10. doi: 10.1186/s40813-024-00358-w (PMC10874036; doi:10.1186/s40813-024-00358-w)
Supplement: Supplementary file 3 — Additional file 3: Fig. S1. Age distribution of pigs examined for umbilical outpouchings grouped by treatment group. Figure S1 shows the age distribution of the examined pigs along with an explanation [file 40813_2024_358_MOESM3_ESM.docx]

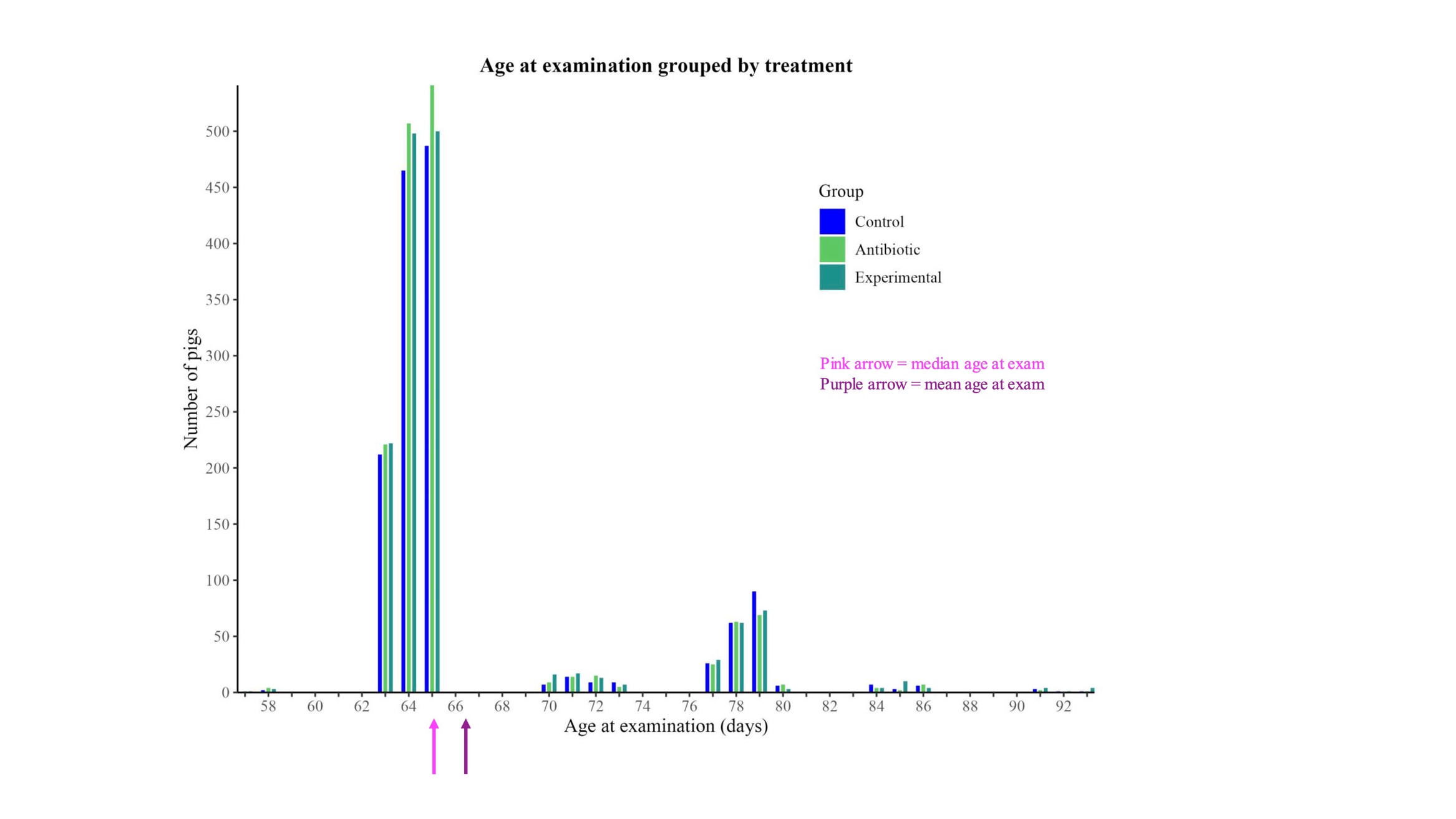


Figure S1 Age distribution of pigs examined for umbilical outpouchings grouped by treatment group

Figure S1 shows the age distribution of the examined pigs.

Pigs following the desired flow in the stable were 63-65 days old at examination. Piglets weaned a week later was examined when they were 70-72 days old. The peak at 77-79 days is because the smallest pigs from each week batch are housed in “baby stables” for two weeks and then they are moved to ordinary stables and follow pigs two weeks younger.

The median age at examination was 65 days and the mean age was 66.4 days.
