# Supplementary material for: Prevention of umbilical outpouchings and mortality in pigs: Meloxicam, tying, cutting, and chlorhexidine versus amoxicillin or no treatment? A clinical field trial
Source: Porcine Health Manag. 2024 Feb 16;10:10. doi: 10.1186/s40813-024-00358-w (PMC10874036; doi:10.1186/s40813-024-00358-w)
Supplement: Supplementary file 4 — Additional file 4: Fig. S2. Piglet´s age at death grouped by cause of death. Figure 3 shows how old the pigs were when they died, and why they died [file 40813_2024_358_MOESM4_ESM.docx]

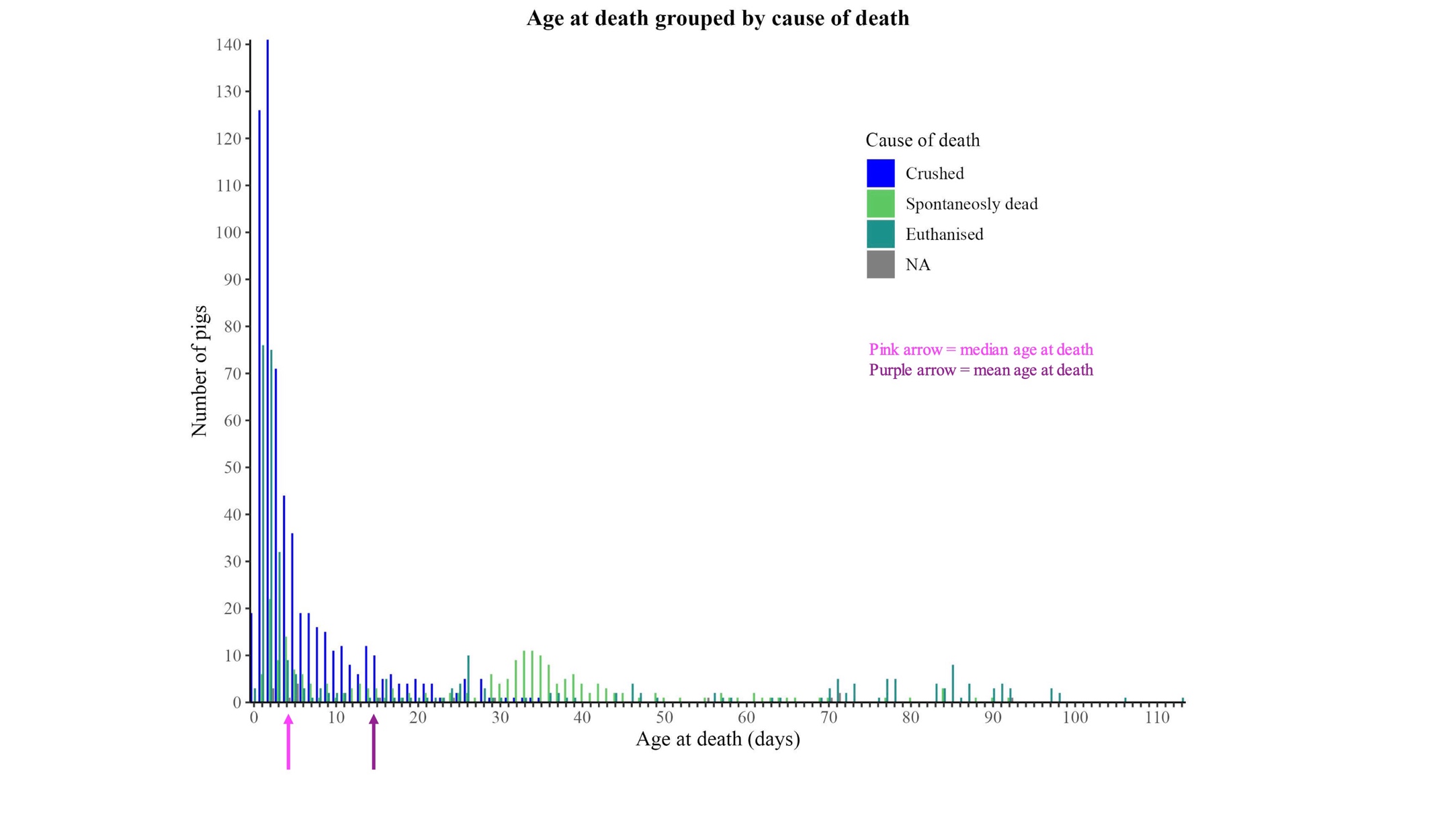
Figure S2 Piglet´s age at death grouped by cause of death

The median age at death was 4 days, and those early deaths were primarily caused by crushings and euthanasias (weak and starved pigs). The peak in dead pigs around 30-40 days was mainly caused by weaning diarrhoea and the euthanasias after 70 days are mainly pigs with UO.

The mean age at death was 14.6 days.
